# Case for diagnosis. Multiple infiltrated plaques in a patient with human immunodeficiency virus and hepatitis C co-infection: lichen myxedematosus^⋆^

**DOI:** 10.1016/j.abd.2021.10.017

**Published:** 2023-02-24

**Authors:** Nathalia Hoffmann Guarda, Renan Rangel Bonamigo, Renata Heck

**Affiliations:** a Sanitary Dermatology Outpatient Clinic, Secretaria de Saúde do Estado do Rio Grande do Sul, Porto Alegre, RS, Brazil; a Sanitary Dermatology Outpatient Clinic, Secretaria de Saúde do Estado do Rio Grande do Sul, Porto Alegre, RS, Brazil; b Faculty of Medicine, Universidade Federal do Rio Grande do Sul, Porto Alegre, RS, Brazil; a Sanitary Dermatology Outpatient Clinic, Secretaria de Saúde do Estado do Rio Grande do Sul, Porto Alegre, RS, Brazil

Dear Editor,

This report describes the case of a 45-year-old male patient, smoker, diagnosed with human immunodeficiency virus (HIV) and hepatitis C virus (HCV) infection three years before. He was undergoing regular treatment with antiretroviral therapy (ritonavir, tenofovir and atazanavir), and had an undetectable viral load, with a CD4 cell count of 534 cells/mm^3^, but without treatment for hepatitis C. He complained of cutaneous lesions with two years of evolution, and significant worsening in the last months, with local pruritus. On examination, erythematous, infiltrated papules and plaques were observed in the gluteal region bilaterally, as well as in the left abdominal, cervical and upper dorsal regions (Figure 1, Figure 2, Figure 3). He underwent laboratory tests that showed AST (aspartate aminotransferase) of 82 U/L, ALT (alanine aminotransferase) of 115 U/L, GGT (gamma-glutamyl transferase) of 131 U/L, alkaline phosphatase of 83 U/L, total bilirubin of 1.28 mg/dL, and fasting glucose of 103 mg/dL. Other laboratory tests within normal limits included: Hb,15.3 g/dL; leukocytes, 5800 mm^3^; platelets, 205,000 mm^3^; TSH, 2.51 IU/mL; free T4 1.08 µg/dL; Cr 0.79 mg/dL; non-reactive ANA (antinuclear antibody), non-reactive rheumatoid factor, proteinogram with no monoclonal peaks. A skin biopsy was performed, which showed abundant mucin deposits in the upper and middle dermis (Fig. 4).

**Figure 1:**
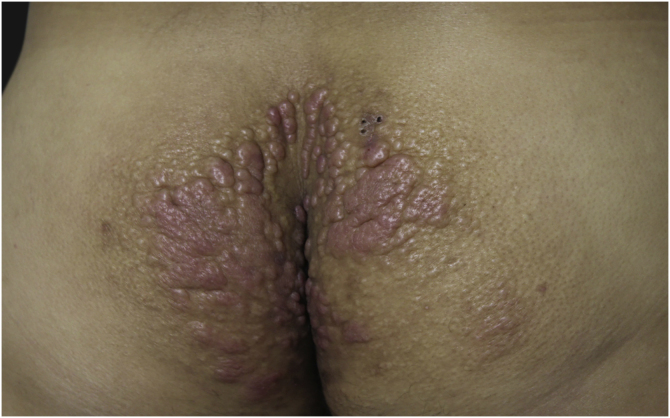
Erythematous, confluent plaques with an infiltrated appearance, in the gluteal region, bilaterally.

**Figure 2:**
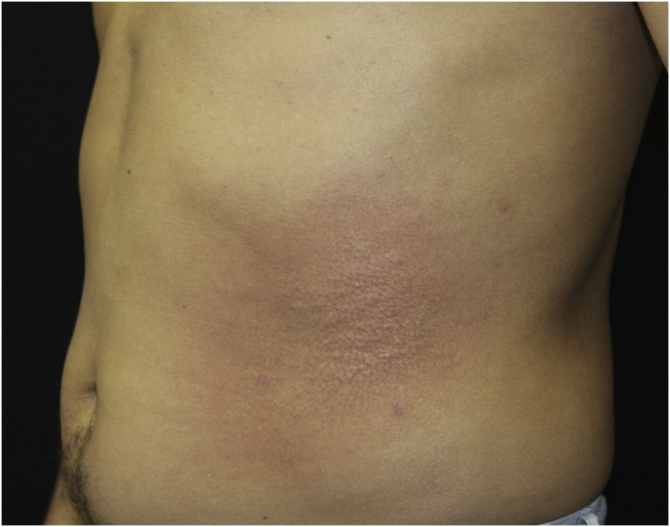
Erythematous plaque with a papular center on the left abdominal region.

**Figure 3:**
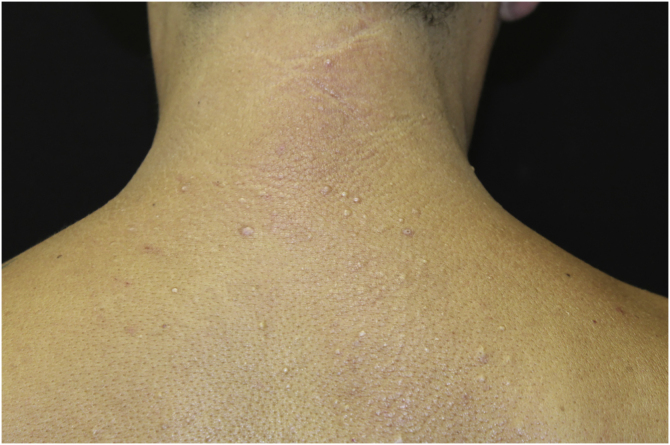
Erythematous, whitish papules on the upper back and posterior cervical regions.

**Figure 4:**
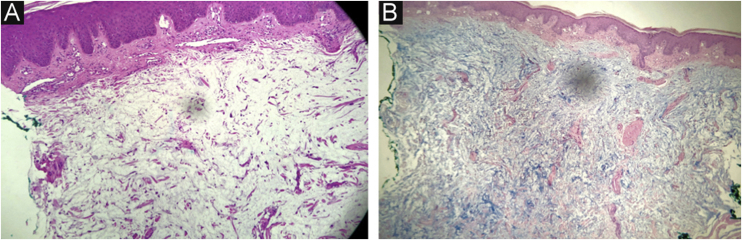
**(A)** Mucin deposits in the upper and middle dermis, with no associated fibroblastic proliferation; absence of amyloid (Hematoxylin & eosin, ×40). (B) Special staining highlights mucin deposition in the dermis (Alcian Blue ×40).

## What’s your diagnosis?

a) Lichen amyloidosus;
b) Lichen myxedematosus;
c) Eruptive collagenoma;
d) Granuloma annulare.

## Discussion

The diagnosis of lichen myxedematosus was confirmed through clinical-pathological correlation. The patient was instructed to maintain antiretroviral therapy and to start treatment for hepatitis C.

Lichen myxedematous (LM) is a rare, chronic subtype of mucinosis that clinically manifests as papules, nodules, or plaques restricted to the skin. It is characterized by fibroblast proliferation, with varying degrees of fibrosis, and mucin deposition in the dermis, in the absence of thyroid disease.^1^, ^2^, ^3^ Its etiopathogenesis is unknown; however, it is known that a variety of clinical conditions have been associated with LM, such as HIV infection, HCV, and exposure to chemicals such as L-tryptophan.^2^, ^3^, ^4^, ^5^, ^6^, ^7^

The current classification of mucinoses was proposed by Rongioletti et al. and divides the papular mucinoses into scleromyxedema, a variant with systemic involvement and associated with paraproteinemia, and localized papular LM. Localized LM is divided into 5 subtypes: discrete papular mucinosis, persistent acral papular mucinosis, self-healing cutaneous mucinosis, juvenile papular mucinosis, and nodular papular mucinosis.^1^, ^6^ Atypical cases with the overlapping of subtypes and distinct characteristics may occur.^1^, ^6^ The patient in the present case can be classified as having localized papular LM of the mild papular mucinosis subtype.

Diagnostic criteria include papular rash, mucin deposition, and variable degree of fibroblast proliferation on histopathological examination, as well as the absence of gammopathy, thyroid disease, or systemic involvement.^4^ Histopathology shows mucin deposition, predominantly in the middle and upper dermis.^3^ The differential diagnosis of LM includes granuloma annulare, lichen amyloidosus, lichenoid eruptions, lichen planus, and eruptive collagenoma.^4^

There are no well-defined treatments reported in the literature, and the recommended approach is clinical observation alone.^3^, ^4^ In general, the prognosis is good, even without specific treatment, and in rare cases, spontaneous resolution may occur.^3^, ^8^ To date, there is no description of the evolution of localized conditions to scleromyxedema. Topical corticosteroids and calcineurin inhibitors are used to relieve symptoms.^3^

## Financial support

None declared.

## Authors’ contributions

Nathalia Hoffmann Guarda: Design and planning of the study; drafting and editing of the manuscript; collection, analysis, and interpretation of data; effective participation in research orientation; intellectual participation in the propaedeutic and/or therapeutic conduct of the studied cases; critical review of the literature; critical review of the manuscript.

Renan Rangel Bonamigo: Approval of the final version of the manuscript; design and planning of the study; effective participation in research orientation; intellectual participation in the propaedeutic and/or therapeutic conduct of the studied cases; critical review of the literature; critical review of the manuscript.

Renata Heck: Approval of the final version of the manuscript; design and planning of the study; effective participation in research orientation; intellectual participation in the propaedeutic and/or therapeutic conduct of the studied cases; critical review of the literature; critical review of the manuscript.

## Conflicts of interest

None declared.
